# Modeling the temporal dynamics of cervicovaginal microbiota identifies targets that may promote reproductive health

**DOI:** 10.1186/s40168-021-01096-9

**Published:** 2021-07-26

**Authors:** Alexander Munoz, Matthew R. Hayward, Seth M. Bloom, Muntsa Rocafort, Sinaye Ngcapu, Nomfuneko A. Mafunda, Jiawu Xu, Nondumiso Xulu, Mary Dong, Krista L. Dong, Nasreen Ismail, Thumbi Ndung’u, Musie S. Ghebremichael, Douglas S. Kwon

**Affiliations:** 1 Ragon Institute of MGH, MIT, and Harvard, 400 Technology Square, Cambridge, MA 02139 USA; 2 Harvard Medical School, Boston, MA 02115 USA; 3 Division of Infectious Diseases, Massachusetts General Hospital, Boston, MA 02114 USA; 4 Centre for the AIDS Programme of Research in South Africa (CAPRISA), Doris Duke Medical Research Institute, Nelson R Mandela School of Medicine, University of KwaZulu-Natal, Durban, South Africa; 5 Department of Medical Microbiology, University of KwaZulu-Natal, Durban, South Africa; 6 HIV Pathogenesis Programme (HPP), Doris Duke Medical Research Institute, Nelson R Mandela School of Medicine, University of KwaZulu-Natal, Durban, South Africa; 7 Females Rising through Education, Support, and Health, Durban, KwaZulu-Natal South Africa; 8 Massachusetts General Hospital, Boston, MA 02114 USA; 9 Africa Health Research Institute (AHRI), Durban, South Africa; 10 Max Planck Institute for Infection Biology, Berlin, Germany; 11 Division of Infection and Immunity, University College London, London, United Kingdom

## Abstract

**Background:**

Cervicovaginal bacterial communities composed of diverse anaerobes with low *Lactobacillus* abundance are associated with poor reproductive outcomes such as preterm birth, infertility, cervicitis, and risk of sexually transmitted infections (STIs), including human immunodeficiency virus (HIV). Women in sub-Saharan Africa have a higher prevalence of these high-risk bacterial communities when compared to Western populations. However, the transition of cervicovaginal communities between high- and low-risk community states over time is not well described in African populations.

**Results:**

We profiled the bacterial composition of 316 cervicovaginal swabs collected at 3-month intervals from 88 healthy young Black South African women with a median follow-up of 9 months per participant and developed a Markov-based model of transition dynamics that accurately predicted bacterial composition within a broader cross-sectional cohort. We found that *Lactobacillus iners*-dominant, but not *Lactobacillus crispatus*-dominant, communities have a high probability of transitioning to high-risk states. Simulating clinical interventions by manipulating the underlying transition probabilities, our model predicts that the population prevalence of low-risk microbial communities could most effectively be increased by manipulating the movement between *L. iners*- and *L. crispatus*-dominant communities.

**Conclusions:**

The Markov model we present here indicates that *L. iners*-dominant communities have a high probability of transitioning to higher-risk states. We additionally identify transitions to target to increase the prevalence of *L. crispatus*-dominant communities. These findings may help guide future intervention strategies targeted at reducing bacteria-associated adverse reproductive outcomes among women living in sub-Saharan Africa.

**Video Abstract**

**Supplementary Information:**

The online version contains supplementary material available at 10.1186/s40168-021-01096-9.

## Background

The cervicovaginal microbiome impacts several important reproductive outcomes, including preterm birth [1, 2], fertility [3], cervicitis [4, 5], and risk of sexually transmitted infections (STIs) [6], including human immunodeficiency virus (HIV) [7–10]. We previously defined four discrete bacterial community assemblages, or cervicotypes (CTs), in the female genital tract (FGT) microbiome of a cohort of healthy Black South African women of reproductive age [11]. Approximately 60% of women in this cohort have diverse bacterial communities with low abundance of *Lactobacillus* species that can be classified into one of two CTs—CT3, which is dominated by *Gardnerella vaginalis*, and CT4, which features diverse taxa but is generally *Prevotella*-rich. The remainder of women in this cohort has low-diversity, *Lactobacillus*-dominant microbial communities dominated by either *Lactobacillus crispatus* (CT1, ~10% of total population) or *Lactobacillus iners* (CT2, ~30% of total population). The low population frequency of *L. crispatus*, which has been replicated in other sub-Saharan African cohorts [7, 12, 13], stands in marked contrast to findings among Caucasian women in the U.S. and Europe, where 80–90% of women have FGT microbiota dominated by *Lactobacillus* species with a higher prevalence of *L. crispatus*-dominated communities than of *L. iners* [14–16]. Importantly, CT3 and CT4 communities are highly correlated with genital pro-inflammatory cytokine concentrations when compared to *Lactobacillus*-dominated communities. In addition, CT4 is associated with higher numbers of activated cervical CD4+ T cells (i.e., HIV target cells) and an over four-fold higher risk of HIV acquisition [11]. Notably, high diversity cervicovaginal communities with low *Lactobacillus* abundance are a characteristic feature of clinical bacterial vaginosis (BV), a common condition associated with vaginal symptoms such as discharge, malodor, and discomfort [17]. BV can be treated with antibiotics, but treatment frequently fails to durably establish a low-diversity, *Lactobacillus*-dominated state [18, 19] that appears optimal for reproductive health.

While cross-sectional surveys provide valuable insight into FGT bacterial population structure and disease associations, they fail to capture important information about temporal changes in bacterial composition. Longitudinal studies performed in high-resource countries have shown that cervicovaginal communities can be dynamic, with composition shifting between community state types over the span of days to weeks [20–24]. No study to date has used sequencing-based approaches to model temporal dynamics of the FGT microbiota in women living in sub-Saharan Africa, where diverse microbial communities predominate and where the burden of adverse reproductive outcomes associated with these microbial communities is greatest. We hypothesized that longitudinally characterizing FGT bacterial composition in a representative sub-Saharan African cohort would allow us to model the dynamics that shape population-level prevalence patterns and identify potential targets for interventions to promote low-risk microbial communities.

In order to elucidate the longitudinal dynamics of FGT bacterial composition, we performed culture-independent bacterial 16S rRNA gene sequencing and bacterial community profiling on cervical swabs collected at 3-month intervals from a cohort of healthy, HIV-uninfected, Black South African women, and classified them into CTs. We constructed a Markov-based model of CT transitions and performed Monte Carlo simulation and parameter sensitivity analyses to predict interventions that could effectively shift CT stationary distributions from high-inflammatory CT3 and CT4 to low-inflammatory CT1. We found that CT2 (*L. iners*-dominant) acted as a gateway to more diverse and stable CT3 and CT4 communities, which were previously associated with elevated genital inflammation [25]. Interestingly, we found that direct transitions between CT1 and CT3 or CT4 were rare. Monte Carlo simulation and parameter sensitivity analyses predicted that blocking the transition from CT1 to CT2 and boosting the transition from CT2 to CT1 would be the most effective intervention to increase population prevalence of *L. crispatus*-dominant communities (CT1), which are associated with lower levels of mucosal inflammation and more favorable reproductive health.

## Results

### Markov-based model of cervicovaginal microbiota temporal dynamics identifies transient and persistent states

In order to characterize the longitudinal dynamics of FGT microbiota, we collected cervicovaginal swab samples at 3-month intervals from 88 healthy, HIV-uninfected, Black South African women enrolled in the FRESH (Females Rising Through Educational Support and Health) cohort [26]. Participants had a median age of 21 years (range 18 to 24 years) at the time of initial sample collection, and baseline survey data on sexual-behavioral and vaginal hygiene practices were available for 86 of the 88 participants (Supplementary Table 1). Forty-four participants (50%) reported currently being on some form of hormonal or long-acting contraceptive method at baseline, with the majority on injectable hormonal contraception including depot-medroxyprogesterone acetate (30.7%) or norethisterone enantate (9.1%). Sixty-six participants (75%) reported sex within the past 30 days, with a median of two sexual encounters per participant; none reported having more than one partner during that interval. Of the 66 participants reporting recent sexual activity, 24 (27.3%) reported always using condoms, 30 (34.1%) reported sometimes using condoms, and 12 (13.6%) reported never using condoms. Although antibiotic usage information was not collected at the time these participants were enrolled, analysis of 1444 sampling encounters within the study occurring after institution of antibiotic usage surveys indicated that self-reported antibiotic usage was rare, with only 3.46% of encounters reporting antibiotic treatment in the preceding 30 days.

**Fig. 1:**
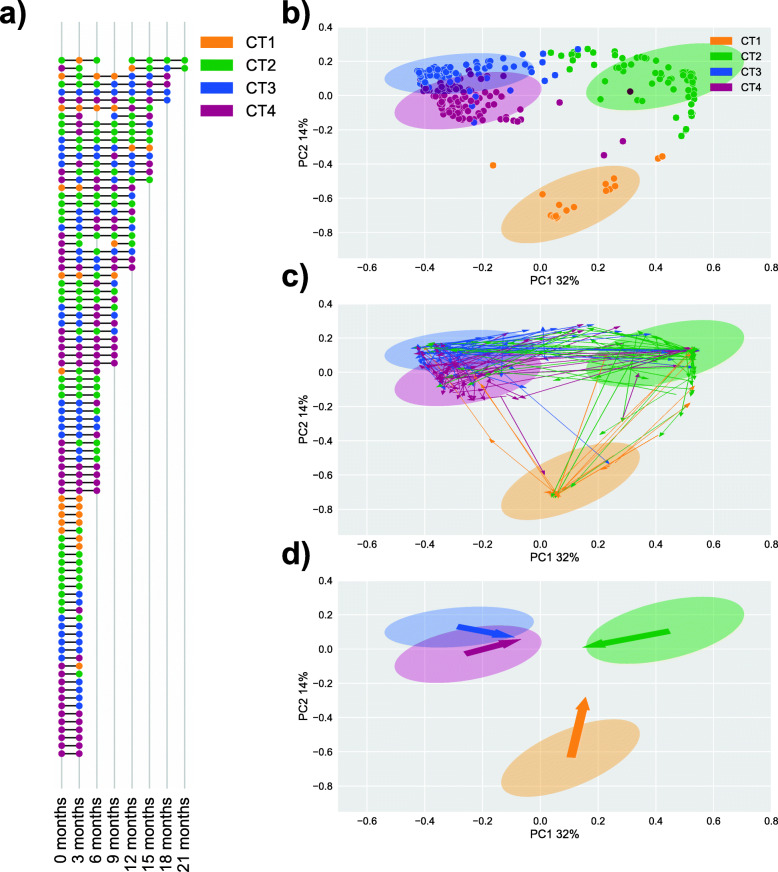
**A** Cervicotype (CT) assignments were given to 316 samples taken from 88 women. Each row represents the cervicotype designation for each sample in a time series of a given woman. Points are joined when adjacent samples are separated by a 3-month interval. **B**–**D** Principle coordinate analysis (PCoA) of Bray-Curtis dissimilarity of species-level relative abundances. Samples are colored by CT assignment with ellipses representing an interval of 2 standard deviations around the centroid of a given cluster. **B** Points represent samples projected in to the PCoA space. **C** Adjacent time points separated by a 3-month interval are connected by an arrow colored by the CT designation of the early time point. **D** Summing the vectors by the CT designation of the earlier timepoint shows the overall direction of movement of samples beginning in a particular CT

Longitudinal sampling covered a median follow-up duration of 9 months per participant (range 3–21 months) with a median of 3 samples per participant (range 2–7 samples) for a total of 316 samples (Fig. 1A). The microbial composition of the samples was characterized through bacterial 16S rRNA gene sequencing, and bacterial communities were classified into cervicotypes (CTs) as previously described [11].

To visualize transitions between CTs, we performed principal coordinate analysis (PCoA) on Bray-Curtis dissimilarity of species-level relative abundances (Fig. 1B). Using arrows to represent transitions between adjacent timepoints for a participant (excluding any transitions that spanned missing timepoints), we observed numerous movements in PCoA space over time, demonstrating the dynamic nature of these communities, with many transitions from low-diversity to high-diversity CTs (Fig. 1C, Suppl. Fig. 1). By taking the vector sum of arrows leaving each CT, we determined the net movement in PCoA space starting from a given CT (Fig. 1D). This demonstrated that individuals with CT1 communities had the tendency to transition to CT2, while CT2 communities moved toward CT3 and CT4 states. Direct transitions between CT1 and CT3 or CT4 were rarely observed and women with CT3 or CT4 communities tended to stay within these diverse states (Suppl Fig. 1 and 2; Suppl. Video 1).

**Fig. 2:**
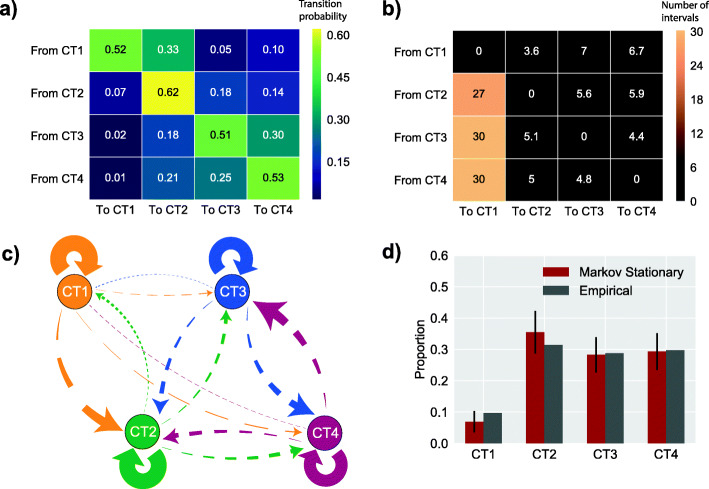
**A** Transition probabilities of moving from one CT to another after a 3-month interval. **B** The mean simulated 3-month time steps required to move between CTs. **C** Markov model diagram of transition states over the CT space where arrow thickness is proportional to the probability of that transition occurring. Number of breaks in the arrow reflects the number of 3-month time steps required to move between community states. **D** The proportions of the population in each CT at equilibrium state of the Markov chain model is not significantly different to an empiric distribution observed in a prior study from the same cohort (Cressie-Read power divergence statistic = 2.90, p value = 0.407)

We next implemented a discrete-time stochastic model for probabilistic forecasting over the CT state space in order to better understand the longitudinal transitions between communities. To force tractability of the model, we assumed memorylessness—namely, the model was conditional on the present CT where the future and past states were independent, an approach that has previously been used to model dynamics of vaginal bacteria in Western populations [1, 24]. These properties allowed us to apply a finite-state-space, time-homogenous, discrete-time Markov chain model to our data. Each state in the Markov chain was one of the four CTs, and each discrete timestep represented a 3-month interval between sample collection. Examining the transition probabilities (Fig. 2A), we saw that CT1 had a high probability of remaining (0.52) or transitioning to CT2 (0.33), whereas the probability of transitioning to CT3 or CT4 was much lower at 0.05 and 0.10, respectively. Like CT1, CT2 had a high probability of remaining (0.62), although CT2 communities had a higher probability of transitioning to CT3 and CT4 (0.18 and 0.14, respectively). Furthermore, the probability of movement to CT1 was low at 0.07, whereas the movement from CT1 to CT2 was much higher at 0.33. For CT3 and CT4, there was a high probability of remaining in these highly diverse community states (summed probability of 0.81 and 0.78, respectively). Both CT3 and CT4 had low probabilities of transitioning to CT2 (0.18 and 0.21, respectively) and rarely transitioned to CT1 (0.02 and 0.01, respectively).

To further explore the adherence to each CT, we calculated the mean number of timesteps required to transition between each pair of CTs (Fig. 2B). We found that it took 7 and 6.7 steps to transition from CT1 to CT3 or CT4, respectively. However, it took 30 steps to move in the reverse direction, from CT3 or CT4 to CT1. Unlike CT1, the transition from CT3 or CT4 back to CT2 took just 5 times steps. The largest disparity in directionality was between CT1 and CT2, with CT1 to CT2 transition taking just 3.6-time steps while the reverse took 27.

Overall, analyses performed on the Markov model indicate that *L. iners-*dominant CT2 acts as an intermediary or “gateway” state between *L. crispatus*-dominant CT1 communities, which are most strongly associated with favorable reproductive outcomes, and the more diverse and higher-risk communities, CT3 and CT4 (Fig. 2C).

Having established that the Markov chain model shows differences in community adherence, we assessed whether the model could predict an empiric distribution of CTs within the same population. To do this, we calculated the stationary distribution of the transition probability matrix. The median CT distribution predicted by this calculation was not significantly different from a previously reported empirical CT distribution of a large cross-sectional sample (n = 236 participants) from the same cohort [11] (Fig. 2D; Cressie-Read power divergence statistic = 2.90, p value = 0.407; a power calculation determined that we were able to detect a total probability difference across all CTs of 5% or greater at a p value threshold of 0.05; Suppl Fig. 3). The concordance between simulated and empiric distributions supported the validity of the underlying Markov model and suggested that it could be used to make valid predictions about the effects of different types of interventions on cervicotype distributions within the populations.

### *L. iners*-dominated communities with higher diversity have a greater probability of transitioning

**Fig. 3:**
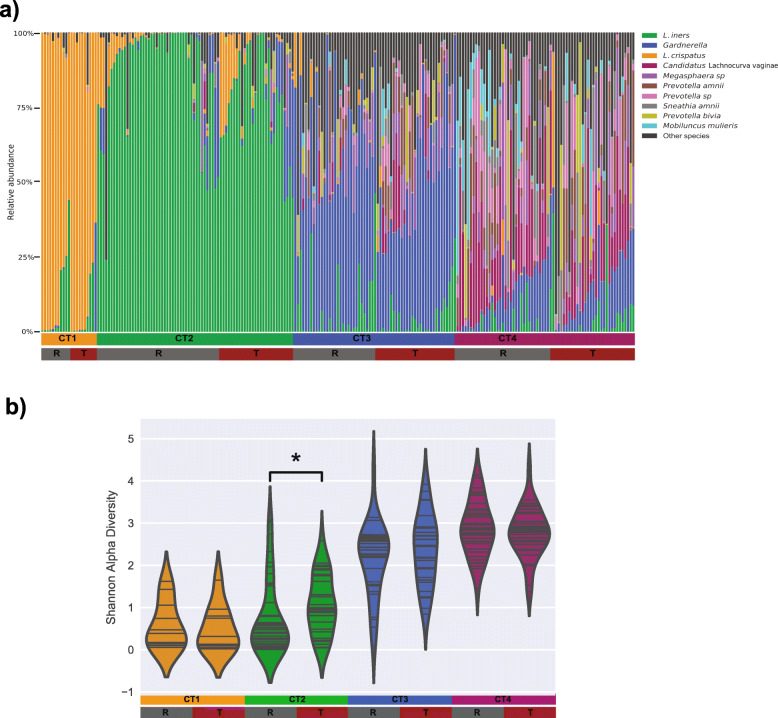
**A** Stacked barplots depicting the microbial composition of 316 samples from 88 women; samples are separated by cervicotype (CT) assignment and subdivided by samples from communities which remain in the same CT (**R**) at the next timepoint and communities that transition to a different CT (**T**). **B** CT2 communities which transitioned to another CT had a higher Shannon alpha diversity than those that remained (non-parametric two-sample Monte Carlo t tests. *p < 0.05)

In order to better understand the community transitions, we compared samples that remained in the same CT at the next timepoint (“Remained”, R) and those that transitioned to a different CT (“Transitioned”, T; Fig. 3A). We observed that the Shannon alpha diversity was significantly higher in CT2 communities which transitioned compared to those that remained (p < 0.05; Fig. 3B). In contrast, CT1, CT2, and CT4 transitions were not associated with differences in Shannon alpha diversity.

### Time-discrete perturbations that establish CT2, CT3, or CT4 dominance revert to the equilibrium state more rapidly than perturbations that establish CT1 dominance

**Fig. 4:**
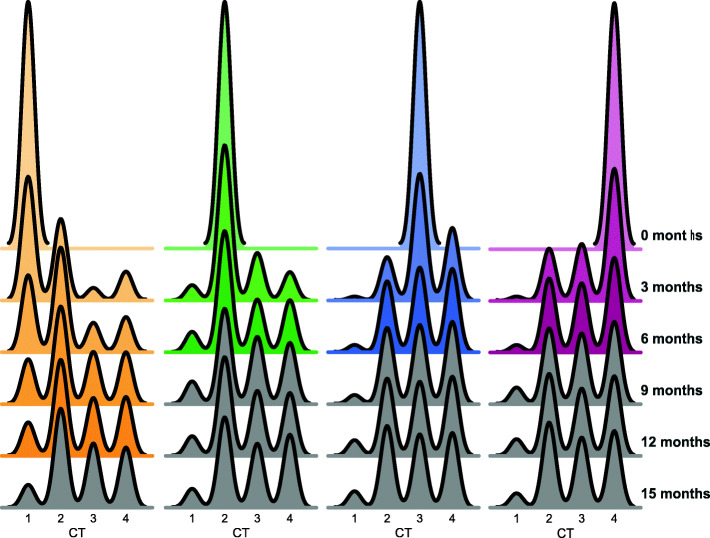
Simulated transition dynamics following a theoretical intervention shifting all individuals in the population to a single CT. CT2, CT3, and CT4 quickly assume the equilibrium distribution whereas CT1 populations have a markedly slower movement (distributions are grayed out when chi-squared p value between the perturbed distribution and the equilibrium distribution of the Markov Model exceeded 0.05)

Treatment of bacterial vaginosis (BV) using *Lactobacillus*-based probiotics or antibiotics such as metronidazole can shift cervicovaginal communities to *Lactobacillus*-dominant states [20, 27, 28], but recurrence rates after BV treatment are high [18, 19, 29, 30]. After demonstrating that transition probabilities between the different CTs can explain an equilibrium state with a high prevalence of diverse, pro-inflammatory communities, we investigated the impact of simulated perturbations at a single timepoint on subsequent CT distribution dynamics. We simulated transition dynamics following a theoretical intervention shifting all individuals in the population to a single CT. We then determined the number of time points it would take to assume the equilibrium distribution of the model by iteratively calculating the chi-squared p value between the number of individuals predicted to be in that state at each simulated timepoint and the equilibrium distribution, defining a return to equilibrium as a p value > 0.05 (Suppl. Fig. 4). A population beginning with CT2, CT3, or CT4 quickly rebounded, reaching the equilibrium distribution after just 3 iterations (Fig. 4). Although CT3 and CT4 communities need to pass through intermediate CT2 to replenish CT1 numbers, the low numbers of CT1 communities in the equilibrium distribution mean this was quickly achieved. Shifting to CT1 slowed the recurrence to the equilibrium state, requiring 5 iterations (Fig. 4), reflecting the additional time taken to move through the CT2 intermediate state to replenish the high numbers of CT3 and CT4 communities. These results show that regardless of the cross-sectional intervention, none are particularly effective at shifting the population CT distribution for long periods; however, cross-sectional interventions shifting to CT1 appear to have more durable effects than other cross-sectional interventions.

### Shifting CT1-CT2 transition probabilities has the largest predicted impact on the prevalence of *L. crispatus*-dominant communities

After demonstrating the transient effects of time-discrete interventions on population-level CT prevalence, we next investigated the impact of changes on the underlying transition probabilities of the chain. Specifically, we modeled the effects of two types of simulated interventions on predicted population equilibrium distributions of CTs: “boosting,” which increases the probability of a directional transition between two CTs, and “blocking,” which reduces the probability of a directional transition.

**Fig. 5:**
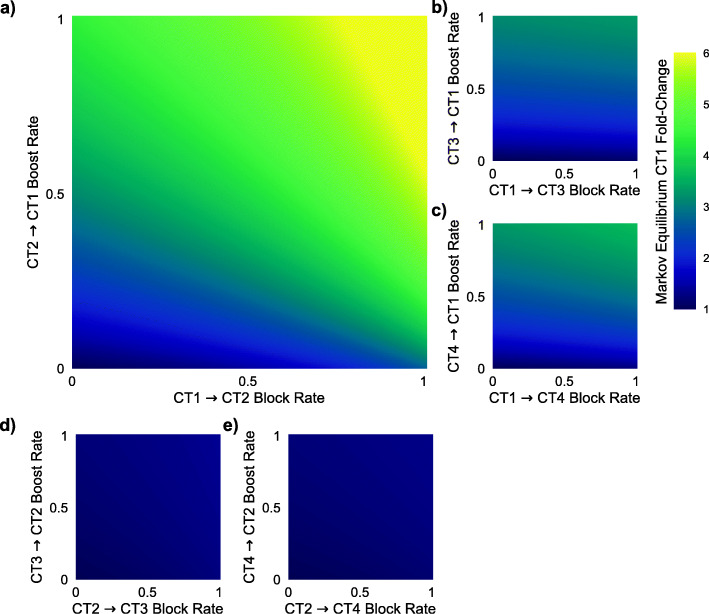
**A**–**E** Predicted fold-change in proportion of population in CT1 at stationary distribution following edge-boost and edge-block interventions along an individual edge (with the indicated boost and block rates) between combinations of high (CT3 and CT4) and low (CT1 and CT2) diversity communities. The most effective intervention to increase CT1 prevalence in the population was to simultaneously boost the transition from CT2 to CT1 and block the transition from CT1 to CT2 (**A**)

We simulated blocking and boosting interventions between high (CT3 and CT4) and low (CT1 and CT2) diversity communities, calculating new CT stationary distributions based on the modified transition probability matrices (Fig. 5). We focused on the fold increase in CT1 population prevalence at equilibrium, as any clinical intervention would ideally increase CT1 prevalence given its association with favorable reproductive outcomes. Corresponding to our previous findings regarding the transient nature of *L. iners* dominance, blocking or boosting transitions between CT2-CT3 and CT2-CT4 had a negligible effect on the proportion of CT1 communities at equilibrium (Fig. 5E, D). The most effective interventions were boosting the transition from CT2 to CT1 and blocking the transition from CT1 to CT2 (Fig. 5A). For example, both boosting and blocking the CT2 to CT1 transition by 50% resulted in a 4.1-fold increase in CT1 communities at equilibrium. However, the same 50% boost and block of CT3 to CT1 transition resulted in only a 2.5-fold increase in CT1 communities. Similarly boosting and blocking CT4 to CT1 transition resulted in a 2.7-fold increase. Boosting and blocking of all other CT pairs had less than a 1.25-fold increase in CT1 communities at equilibrium. These findings indicate that interventions specifically targeting the *L. crispatus* to *L. iners* transition may have the greatest impact on increasing the number of women with low-inflammatory, *L. crispatus*-dominated communities within the population.

## Discussion

The FGT microbiome is associated with adverse reproductive outcomes in sub-Saharan Africa [12]. Although the temporal dynamics of vaginal microbiota have been studied in North American populations [20–23], they have not been explored in sub-Saharan African populations. Extrapolation of observations from North America to sub-Saharan African women has been hindered by differences in both the species comprising the FGT microbiome and differences in the prevalence of various species amongst the respective populations. To address these limitations, we used 16S rRNA gene sequencing to profile the bacterial composition of 316 samples collected at 3-month intervals from 88 participants enrolled in an observational cohort of healthy, HIV-uninfected, Black South African women, and then constructed a Markov model to explore the persistence and transition of the microbiome between four previously described community states. We show that in a South African population, *L. iners* dominated communities (CT2) act as a gateway between *L. crispatus* dominated communities (CT1), previously associated with favorable reproductive outcomes, and stable, high-diversity communities (CT3 and CT4), which have been associated with adverse reproductive outcomes [11]. A similar study conducted in North American populations by Brooks et al. 2017, combining data from multiple longitudinal studies with varying sampling intervals, explored longitudinal FGT community dynamics using a different classification system called community state types (CSTs) [20–22, 24, 31]. Within this definition, *L. iners* is found in high abundance in two CSTs, CST3 and CST9. Under the CT definition described in Anahtar et al. 2015 for our South African population, there is only one community with high abundance of *L. iners*, CT2 [25]. These differences in definition make some comparisons between North American and South African findings challenging. However, through summing of the weekly mean transition probabilities of CST3 and CST9, we can approximate a single North American *L. iners*-dominant state allowing us to compare transition probabilities across populations. This reveals that in North American women the most likely transition from *L. crispatus*-dominated CST1 was to a community dominated by *Lactobacillus jensenii* (CST5; mean daily transition probability of 0.15), a species which is rarely found in women in sub-Saharan Africa. The largest transition probability from CST5 was back to CST1 (mean weekly transition probability of 0.13). The summed transition probability of moving from CST1 or CST5 to *L. iners* dominated communities CST3 and CST9 was lower than in our study at 0.07 and 0.1, respectively. Both CST1 and CST5 had a high probability of remaining (mean daily transition probability of 0.46 and 0.48, respectively). These differences in transition probabilities of community states within North American and South African populations may explain the observed higher degree of diverse communities in South African compared to North American women.

Longitudinal studies have performed sampling over varying intervals, including daily [20], weekly [21, 31], and multiple months [22]. Short-term (daily or weekly) sampling intervals have utility in identifying individual factors such as menses that can cause acute or transient changes in bacterial community state composition. However, short sampling intervals may be less well suited to capture the long-term dynamics of community state transitions within a population. We hypothesized that sampling at a 3-month interval would provide insight into the cumulative, long-term transition dynamics that shape overall prevalence of different community states within a population. In confirmation, the CT transition probabilities identified using our 3-month sampling interval predicted a population equilibrium state that accurately reflected true, empiric CT prevalences determined by a cross-sectional profiling of a larger sub-cohort of participants from the FRESH study. This result suggests that our model based on a 3-month interval accurately captures the multifactorial dynamics which maintain CT distributions in this population.

We simulated the effects of various interventions on shifting population prevalence of different microbial community states. Our modeling predicted that time-discrete interventions (simulating one-time shifts to a particular community state without altering underlying probabilities of transitioning between different states) had relatively transient effects at the population level, with the population returning to its equilibrium distribution within 9 months if shifted to CT2, CT3, or CT4. Shifting to CT1 provided moderately more durable effects, with a return to equilibrium distribution estimated to require 15 months. The longer-lasting effects of shifts to CT1 (*L. crispatus*-dominant) are relevant because standard BV treatment tends to promote dominance by *L. iners* rather than *L. crispatus* [20, 27, 28], which may partially explain the frequency and rapidity of BV relapse after therapy [29, 30]. Our model predicts that the most effective intervention to increase the proportion of South African women with *L. crispatus*-dominant communities would be to simultaneously boost transition from *L. iners-* to *L. crispatus-*dominant communities, while blocking the reverse transition from *L. crispatus-* to *L. iners-*dominant communities. Possible interventions to shift CT2-CT1 transitions may include introduction of a *L. crispatus* probiotic along with approaches to selectively kill or inhibit *L. iners* growth.

Our study had a number of limitations. Cervicotype and CST assignments are a high-level characterization of the microbiota on a population scale and do not fully account for dynamics of individual species or distinguish whether key species such as *L. crispatus* are genuinely absent or simply present at low levels within individual hosts with non-CT1 bacterial communities. It is possible that more subtle species-level dynamics influence community transitions and that an individual with a CT3 or CT4 community from which *L. crispatus* is entirely absent may be less likely to transition to a CT1 community. Additionally, our study examines a relatively culturally and geographically homogeneous population of young Black women in a specific urban township community in KwaZulu-Natal, South Africa. The precise microbiota dynamics we report here may depend on factors that differ from other populations in sub-Saharan Africa and beyond, limiting generalizability. However, we note that the cross-sectional cervicotype distribution we report in this cohort resembles cross-sectional distributions observed among women in numerous other sub-Saharan cohorts in countries including South Africa, Zambia, and Rwanda [7, 12, 32]. Therefore, it is possible that community transition dynamics similar to those we report here may also maintain empiric distributions elsewhere across the continent.

Microbial composition and dynamics can be influenced by a wide variety of factors including sexual and vaginal hygiene practices [7, 33, 34], hormonal and menstrual state (including effects of contraception) [20, 35], pregnancy and childbirth [1, 20], antibiotic exposure [36], smoking [37], genetics [37, 38], and socioeconomic factors, the combined effects of which likely account for geographic and racial/ethnic differences between populations [11, 12, 14–16]. Our study’s strength relies on its analysis of an observational cohort of young women with few major exclusion criteria except HIV infection, major pre-existing illness, or pregnancy [26, 39]. Our Markov model, constructed using longitudinal data from a subset of study participants, predicts a microbiome population equilibrium matching the true prevalence within the larger cohort, suggesting we can accurately model the net effects of unmeasured microbiota-influencing factors within this population and make valid population-level predictions about effects of different types of interventions. Future studies will be needed to assess the relative contribution of individual behavioral, socioeconomic, and biological factors in shaping specific dynamics within this population.

## Conclusions

The findings presented here advance our understanding of the longitudinal dynamics of the cervicovaginal microbiota in sub-Saharan African women. We identified the transient nature of CT2, a microbial community state dominated by *L. iners*, and suggest that interventions which target the transition between CT2 and *L. crispatus*-dominant CT1 will have the greatest impact in shifting populations of South African women towards health-associated, low-inflammatory *L. crispatus*-dominant bacterial communities. Current widely used antibiotic therapies for BV are unlikely to favorably shift the CT1/CT2 balance [40]; thus, our findings identify a role for novel, microbiome-directed therapies to help reduce rates of adverse reproductive outcomes, including HIV acquisition, among women living in sub-Saharan African.

## Materials and methods

### Study cohort and sample collection

The 88 study participants were 18- to 24-year-old Black women recruited between 2013 and 2017 with more than two consecutive samples available separated by 3 months, and this cohort was a sub-population of the larger Females Rising through Education, Support, and Health (FRESH) prospective observational study in Umlazi, South Africa [26]. Exclusion criteria included positive HIV-1 viral load, positive pregnancy test, or other significant medical co-morbidity at enrollment. Longitudinal samples from 88 HIV-uninfected women in the cohort were analyzed, each with between 2 and 7 samples (Fig. 1A). The study protocol was approved by the Biomedical Research Ethics Committee of the University of KwaZulu-Natal and the Massachusetts General Hospital Institutional Review Board (2012P001812/MGH), and all participants provided informed consent. HIV status was assessed by twice-weekly HIV RNA viral load testing on finger-prick blood samples, and all microbial samples analyzed here were from visits at which concurrent viral load testing was negative. Genital specimens including the ectocervical swabs (Catch-All Epicenter) used for microbiome analysis were collected at 3-month intervals. Contraception, medication usage, and sexual and hygiene practices were self-reported via survey questions with results as detailed in Supplementary Table 1.

### Sequencing, processing, taxonomic assignment, and cervicotype assignment

DNA was extracted from the ectocervical swab, and the V4 region of the 16S rRNA gene was amplified and sequenced in an Illumina MiSeq as previously described [25]. DADA2 [41] was used to quality filter, trim, and resolve amplicon sequence variants (ASVs). Initial taxonomic annotation was performed using DADA2 and RDP training set 16; species-level annotations were assigned when an ASV had an exact match to a sequence in the RDP species set 16 (https://benjjneb.github.io/dada2). Taxonomic assignments were further curated using an internally developed taxonomic database (ASV taxonomic assignments can be found in Suppl. file 1).

A cervicotype was assigned to each sample as previously described [11]. In brief, relative abundances of ASVs were summed at species-level where possible and genus-level otherwise. CT1 communities were defined as communities with ≥50% relative abundance of non-*iners Lactobacillus* species. Analysis of all CT1 non-*iners* *Lactobacillus* reads found that >97% were *L. crispatus*. Communities in which *L. iners* or *Gardnerella* species had the highest relative abundance were defined as CT2 or CT3, respectively, while communities in which another taxon was most abundant were assigned to CT4.

### Markov chain implementation

Markov chain stationary distributions were calculated using the inverse iterative method in Pykov (https://github.com/riccardoscalco/Pykov). The average steps required to return to a CT were calculated with the “mean first passage time” (mfpt_to) function in Pykov.

### Markov chain simulation

CT-level Markov Chain simulation was performed by matrix-multiplying the previous timepoint’s CT distribution and the transition probability matrix *Q* to generate a new probability vector over the state space. These probability vectors represent the simulated CT distribution after a 3-month time interval. We thus iteratively multiplied the transition probability matrix *Q* by an artificial vector assigning 100% of samples to each of the four CTs to simulate the time course of returning to equilibrium after a cross-sectional event shifting the entire population to 100% assignment to each of the CTs in 3-month intervals. We define a simulation as having converged to equilibrium if a chi-square test comparing a timepoint of a simulated distribution to the stationary distribution is not statistically significant with p value > 0.05.

PCoA-space Monte Carlo simulations were performed by stochastically moving to a point in PCoA space close to the true next point (with probability decreasing exponentially with distance). Multivariate Gaussian noise was applied to the PCoA coordinates before animation.

### Boost and block interventions

The boost interventions shifts α probability density in the transition matrix from returning to CTb instead of transitioning to CTa as can be seen in the pseudocode below:

$$ boost\left( CTa, CTb,\alpha \right):\kern2em {Q}_{b,b}\leftarrow {Q}_{b,b}-\alpha {Q}_{b,b}\kern2em {Q}_{b,a}\leftarrow {Q}_{b,a}+\alpha {Q}_{b,b} $$

The block intervention shifts Β probability density in the transition matrix from transitioning to CTb to instead returning to CTa as can be again seen in the pseudocode below:

$$ block\left( CTa, CTb,\beta \right):\kern2em {Q}_{a,b}\leftarrow {Q}_{a,b}-\beta {Q}_{a,b}\kern2em {Q}_{a,a}\leftarrow {Q}_{a,a}+\beta {Q}_{a,b} $$

### Statistics

Differences in CT Shannon alpha diversity were calculated using a non-parametric two-sample Monte Carlo t tests. P values are two-sided and are not adjusted for multiple hypothesis testing. Statistical analyses were performed in Python with SciPy [42] and NumPy [43].

## Supplementary Information

**Additional file 1.** Supplementary fig. 1 PCoA of Bray-Curtis dissimilarity of species-level relative abundances of 316 samples from 88 women, split by CT of origin, with arrows depicting the movement of a community between sequential time points.

**Additional file 2.** Supplementary fig. 2 Table showing the number of transitions between CTs observed in the study.

**Additional file 3.** Supplementary fig. 3 Statistical power calculation for identifying a given difference between empiric and equilibrium distributions.

**Additional file 4.** Supplementary fig. 4 The number of iterations taken for the models to pass a p-value significance threshold of 0.05 (chi-sq between perturbed and equilibrium distributions).

**Additional file 5.** Supplementary file 1 Taxonomic assignment to ASVs.

**Additional file 6.** Supplementary movie 1 Monte Carlo simulation showing the possible movement, at a 3-month time interval, of an individual through the PCoA-space displayed in Fig 1b.

**Additional file 7.** Supplementary table 1 Cohort demographics.

## Data Availability

Raw V4 16S data can be found on NCBI BioProject PRJNA730929.
