# Correction to: Modeling the temporal dynamics of cervicovaginal microbiota identifies targets that may promote reproductive health

**DOI:** 10.1186/s40168-021-01171-1

**Published:** 2021-10-15

**Authors:** Alexander Munoz, Matthew R. Hayward, Seth M. Bloom, Muntsa Rocafort, Sinaye Ngcapu, Nomfuneko A. Mafunda, Jiawu Xu, Nondumiso Xulu, Mary Dong, Krista L. Dong, Nasreen Ismail, Thumbi Ndung’u, Musie S. Ghebremichael, Douglas S. Kwon

**Affiliations:** 1 Ragon Institute of MGH, MIT, and Harvard, 400 Technology Square, Cambridge, MA 02139 USA; 2 Harvard Medical School, Boston, MA 02115 USA; 3 Division of Infectious Diseases, Massachusetts General Hospital, Boston, MA 02114 USA; 4 Centre for the AIDS Programme of Research in South Africa (CAPRISA), Doris Duke Medical Research Institute, Nelson R Mandela School of Medicine, University of KwaZulu-Natal, Durban, South Africa; 5 Department of Medical Microbiology, University of KwaZulu-Natal, Durban, South Africa; 6 HIV Pathogenesis Programme (HPP), Doris Duke Medical Research Institute, Nelson R Mandela School of Medicine, University of KwaZulu-Natal, Durban, South Africa; 7 Females Rising through Education, Support, and Health, Durban, KwaZulu-Natal South Africa; 8 Massachusetts General Hospital, Boston, MA 02114 USA; 9 Africa Health Research Institute (AHRI), Durban, South Africa; 10 Max Planck Institute for Infection Biology, Berlin, Germany; 11 Division of Infection and Immunity, University College London, London, United Kingdom

**Correction to: Microbiome 9, 163 (2021)**

**https://doi.org/10.1186/s40168-021-01096-9**

Following the publication of the original article [1], the authors noticed a misspelling on the name of one of the co-authors.

“Musie S. Gheb**er**michael” should read “Musie S. Gheb**re**michael”

The original article has been updated.
