# Supplementary figures and images for: Modeling the temporal dynamics of cervicovaginal microbiota identifies targets that may promote reproductive health
Source: Microbiome. 2021 Jul 26;9:163. doi: 10.1186/s40168-021-01096-9 (PMC8314590; doi:10.1186/s40168-021-01096-9)

**Supp fig. 1**

**a)**

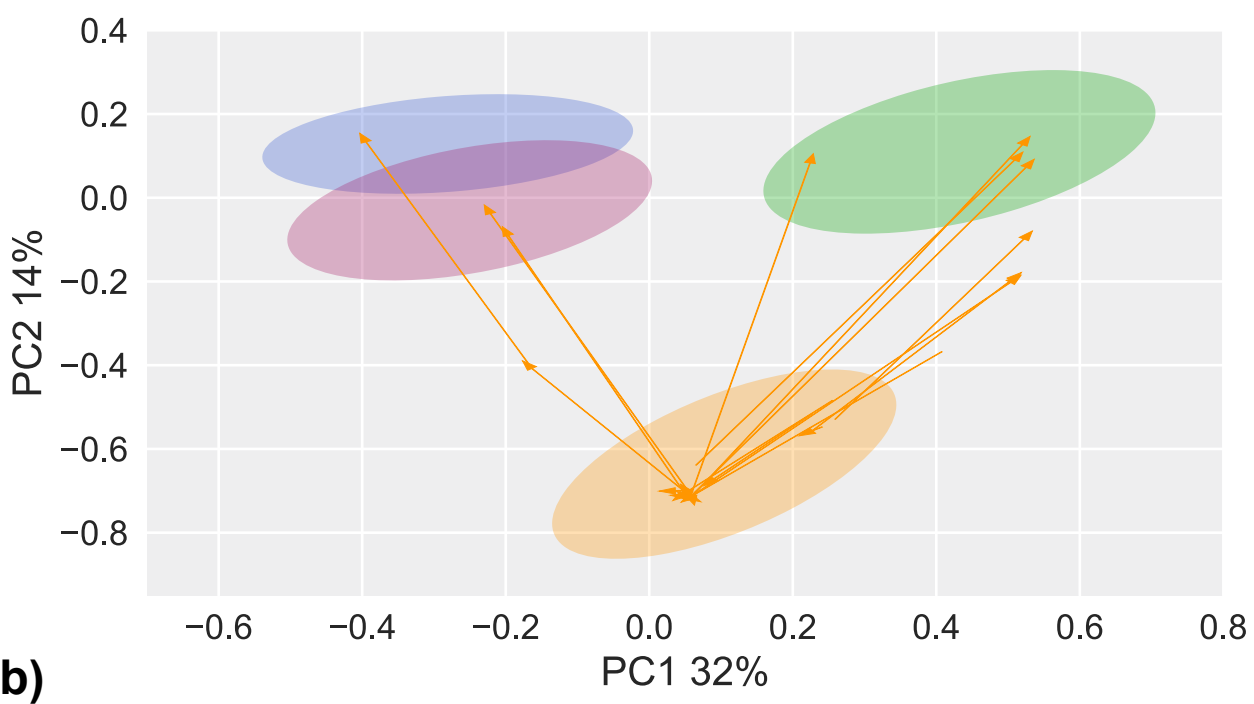

**b)**

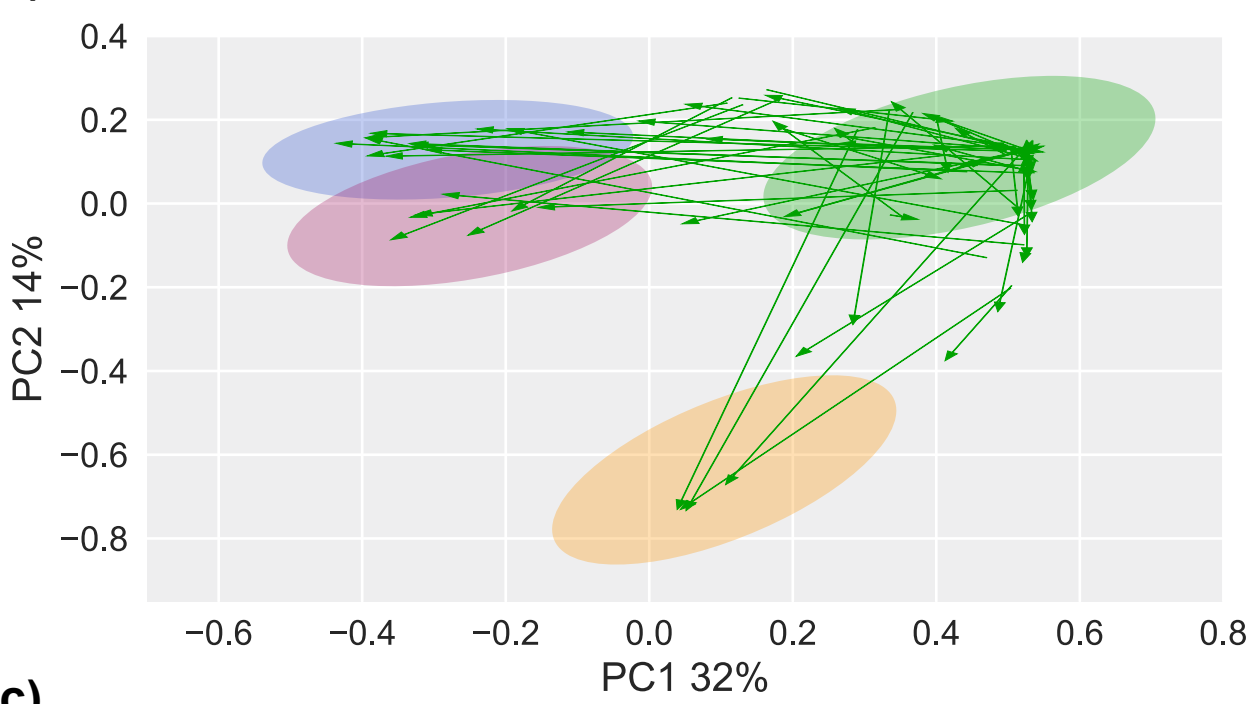

**c)**

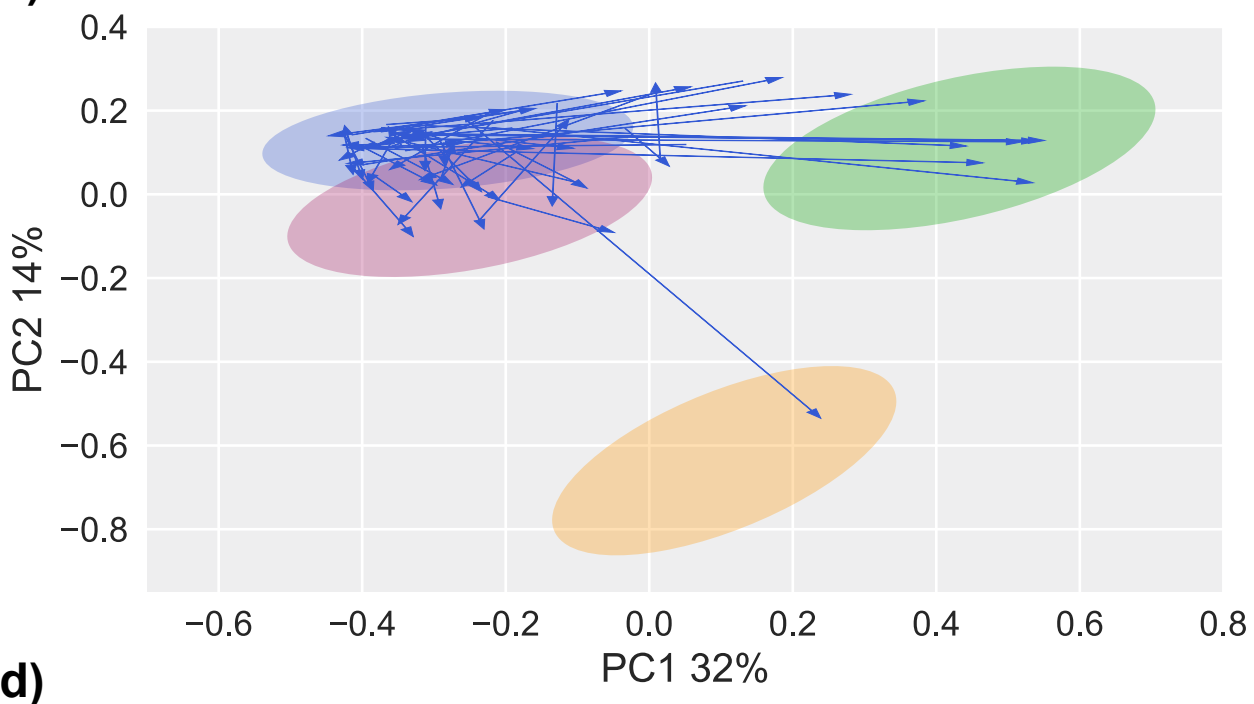

**d)**

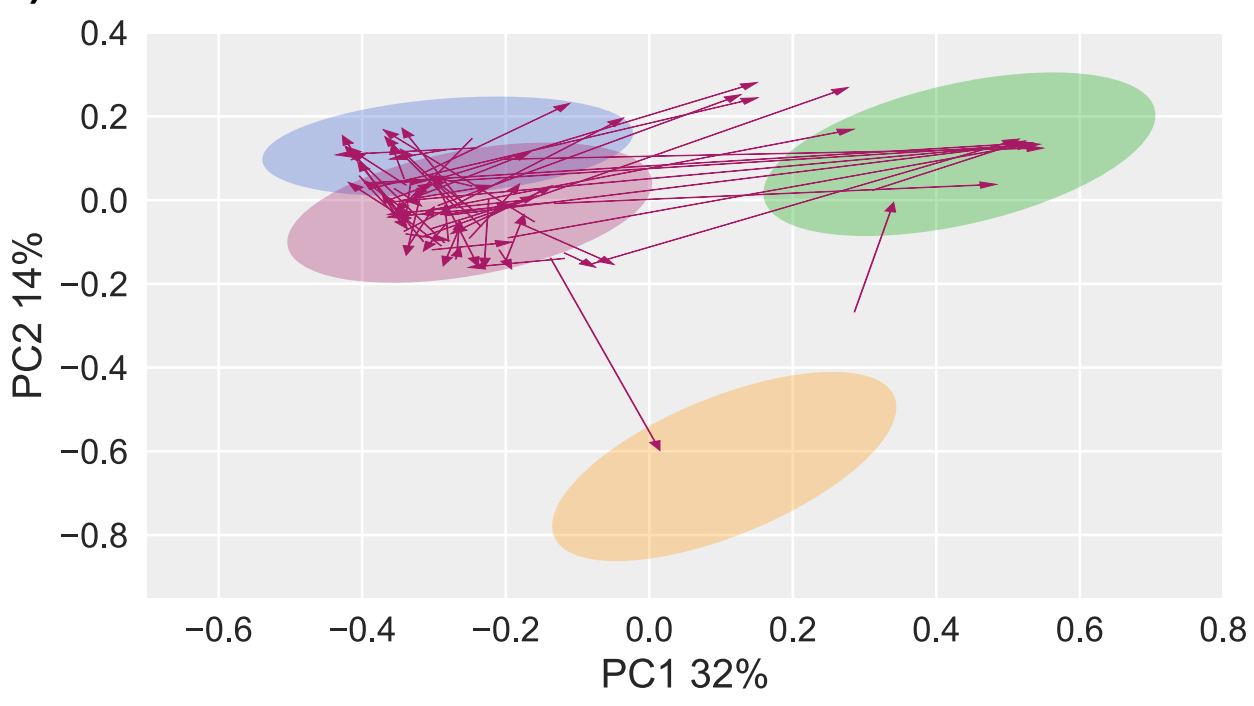

Supplement: Supplementary file 2 — Additional file 1. Supplementary fig. 1 PCoA of Bray-Curtis dissimilarity of species-level relative abundances of 316 samples from 88 women, split by CT of origin, with arrows depicting the movement of a community between sequential time points. [file 40168_2021_1096_MOESM2_ESM.pdf]

**Supp fig. 3**

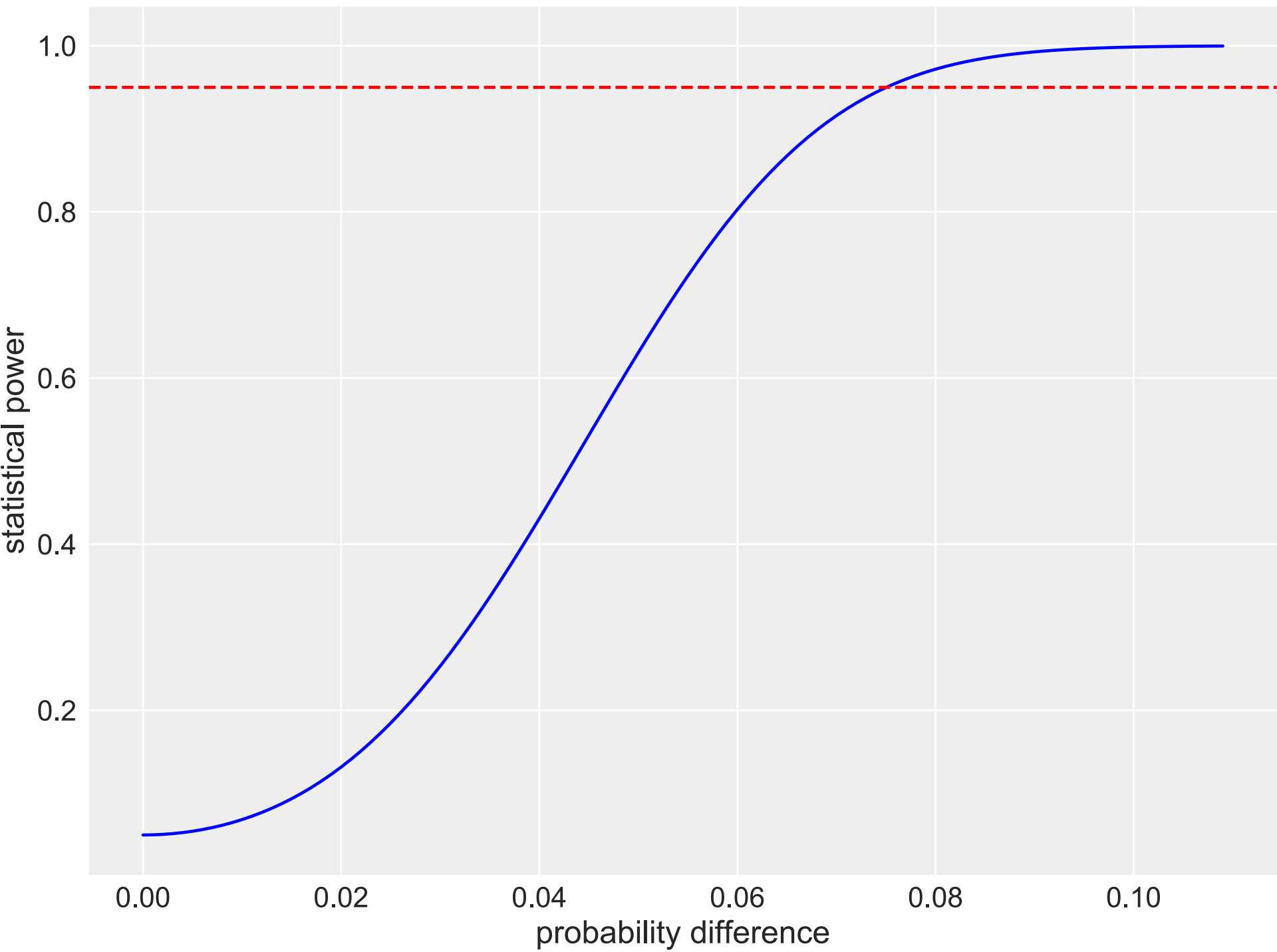

Supplement: Supplementary file 4 — Additional file 3. Supplementary fig. 3 Statistical power calculation for identifying a given difference between empiric and equilibrium distributions. [file 40168_2021_1096_MOESM4_ESM.pdf]

Supp fig. 4

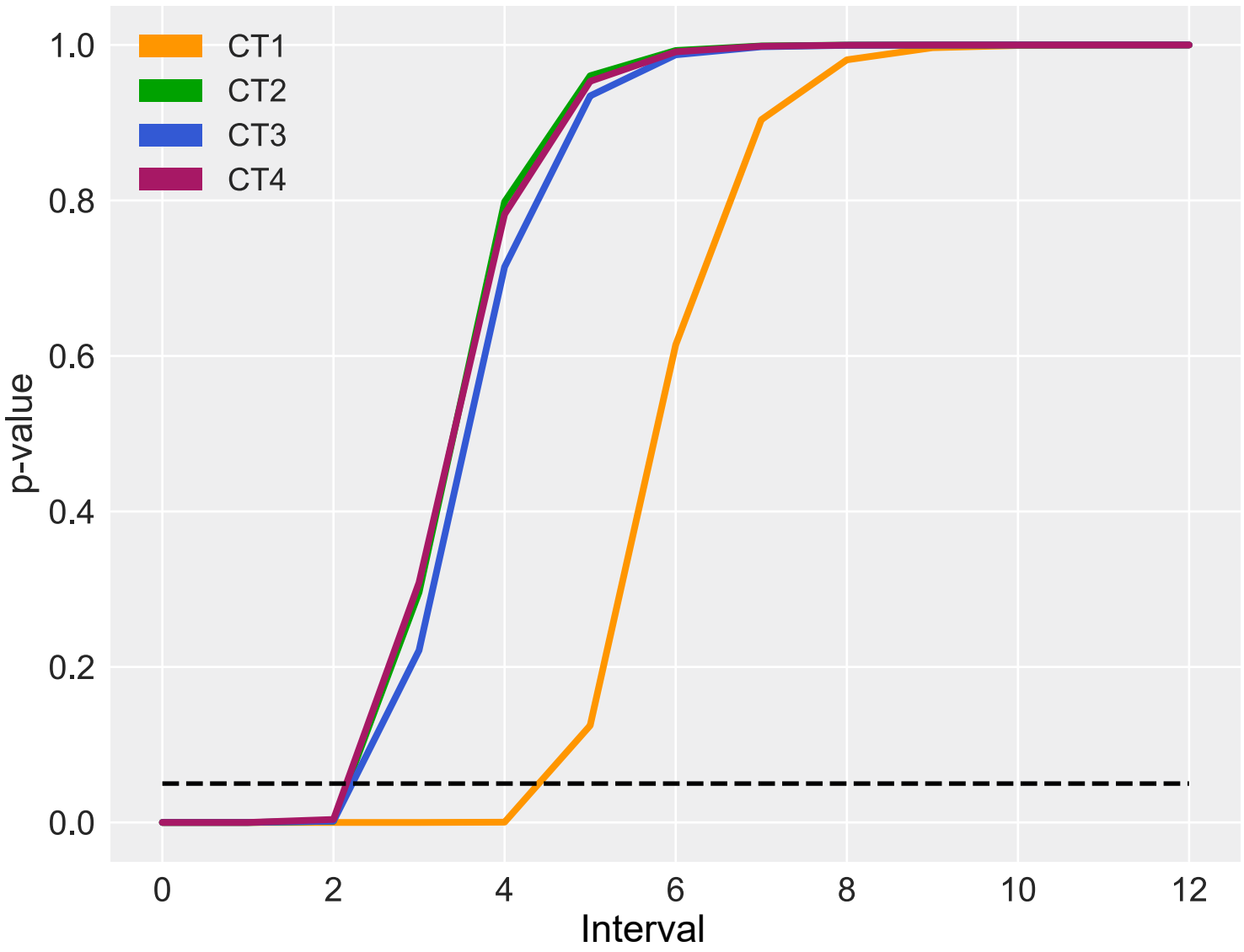

Supplement: Supplementary file 5 — Additional file 4. Supplementary fig. 4 The number of iterations taken for the models to pass a p-value significance threshold of 0.05 (chi-sq between perturbed and equilibrium distributions). [file 40168_2021_1096_MOESM5_ESM.pdf]
