# Supplementary material for: Modeling the temporal dynamics of cervicovaginal microbiota identifies targets that may promote reproductive health
Source: Microbiome. 2021 Jul 26;9:163. doi: 10.1186/s40168-021-01096-9 (PMC8314590; doi:10.1186/s40168-021-01096-9)
Supplement: Supplementary file 3 — Additional file 2. Supplementary fig. 2 Table showing the number of transitions between CTs observed in the study. [file 40168_2021_1096_MOESM3_ESM.pdf]

Supp fig. 2

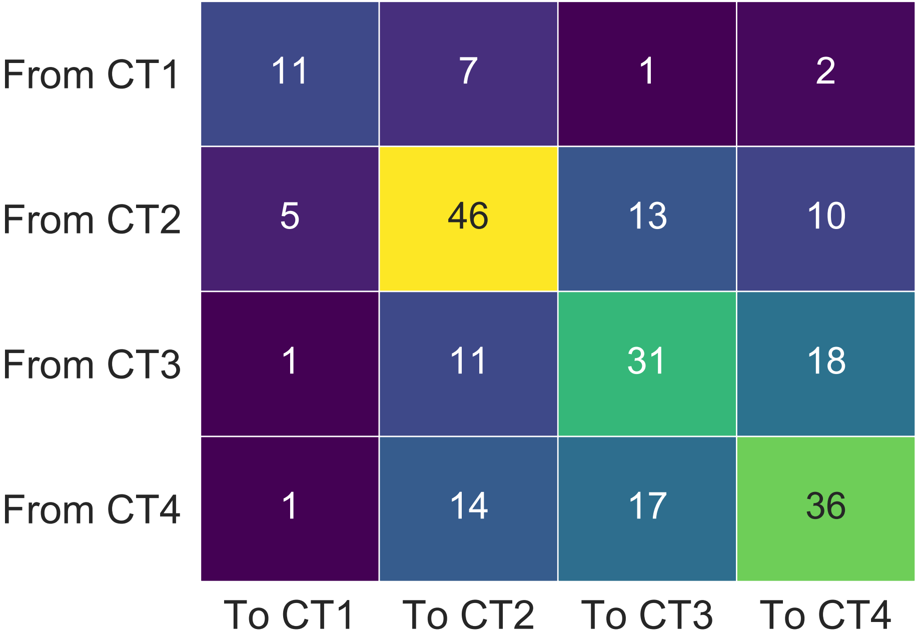
From CT1
11
7
1
2
From CT2
5
46
13
10
From CT3
1
11
31
18
From CT4
1
14
17
36
To CT1
To CT2
To CT3
To CT4
