# Supplementary material for: Modeling the temporal dynamics of cervicovaginal microbiota identifies targets that may promote reproductive health
Source: Microbiome. 2021 Jul 26;9:163. doi: 10.1186/s40168-021-01096-9 (PMC8314590; doi:10.1186/s40168-021-01096-9)
Supplement: Supplementary file 8 — Additional file 7. Supplementary table 1 Cohort demographics. [file 40168_2021_1096_MOESM8_ESM.docx]

**Supplementary Table 1: Participant Demographic and Clinical Characteristics at Time of Collection of First Sequenced Sample**

| **Variable** | **Median**  **(n=88)** | **Range** |
| --- | --- | --- |
| **Age in Years** | 21 | (18 - 24) |
| **Hormonal Contraceptive Method** | **Number** | **%** |
| None | 42 | (47.7%) |
| Depot-medroxyprogesterone acetate (Depo-Provera) | 27 | (30.7%) |
| Norethisterone enantate (Nur-isterate) | 8 | (9.1%) |
| Etonorgestrel implant (Implanon) | 5 | (5.7%) |
| Oral contraceptive pills | 2 | (2.3%) |
| Intra-uterine device | 2 | (2.3%) |
| No data | 2 | (2.3%) |
| **Number of Sex Acts (Past 30 Days)** | **Number** | **%** |
| 0 | 20 | (22.7%) |
| 1-2 | 32 | (36.4%) |
| 3-5 | 28 | (31.8%) |
| 6-10 | 3 | (3.4%) |
| >10 | 3 | (3.4%) |
| No data | 2 | (2.3%) |
| **Number of Sex Partners (Past 30 Days)** | **Number** | **%** |
| 0 | 20 | (22.7%) |
| 1 | 66 | (75%) |
| No data | 2 | (2.3%) |
| **Condom Use (Past 30 Days)** | **Number** | **%** |
| Didn't have sex | 20 | (22.7%) |
| Always | 24 | (27.3%) |
| Sometimes | 30 | (34.1%) |
| Never | 12 | (13.6%) |
| No data | 2 | (2.3%) |
| **Intravaginal Drying/Tightening Agent Use** | **Number** | **%** |
| Never | 69 | (78.4%) |
| Sometimes | 7 | (8%) |
| No data | 12 | (13.6%) |
